# Successful management of a neonate with OTC deficiency presenting with hyperammonemia and severe cardiac dysfunction with extracorporeal membrane oxygenation support and continuous renal replacement therapy

**DOI:** 10.1002/jmd2.12135

**Published:** 2020-06-03

**Authors:** Adrian C. Mattke, Fumiaki Shikata, James McGill, Rob Justo, Prem Venugopal

**Affiliations:** ^1^ Paediatric Intensive Care Unit, Queensland Children's Hospital South Brisbane Queensland Australia; ^2^ Queensland Pediatric Cardiac Service, Queensland Children's Hospital South Brisbane Queensland Australia; ^3^ University of Queensland, Brisbane, School of Medicine St Lucia Queensland Australia; ^4^ Metabolic Medicine, Queensland Children's Hospital South Brisbane Queensland Australia; ^5^ Paediatric Critical Care Research Group, CCHR University of Queensland St Lucia Queensland Australia

**Keywords:** cardiac dysfunction, children, ECMO, extracorporeal, OTC deficiency, renal replacement therapy

## Abstract

Ornithine transcarbamylase (OTC) deficiency is an X‐linked urea cycle disorder which—in severe form—results in rapid accumulation of ammonia and glutamine with subsequent irreversible brain injury. We present a case of severe left ventricular dysfunction with hyperammonemic crisis caused by OTC deficiency which was managed with veno‐arterial extracorporeal membrane oxygenation support combined with continuous renal replacement therapy. Aggressive treatment led to normalization of ammonia and full left ventricular recovery.

**Synopsis**

We report a case of OTC deficiency presenting with severe but reversible cardiac dysfunction that can be treated by extracorporeal membrane oxygenation combined with renal replacement therapy.

## 1 INTRODUCTION

Acute hyperammonemia in a newborn is a medical emergency and rapid treatment is crucial to avoid neurological injury and death.^1^ Ornithine transcarbamylase (OTC) deficiency is an X‐linked urea cycle disorder (UCD) with a reduced ability to convert carbamyl phosphate and ornithine to citrulline which—in neonates—can lead to rapid accumulation of ammonia and glutamine and subsequent acute cytotoxic brain oedema.^1^ Treatment of acute neonatal hyperammonemia includes stopping protein intake, administration of intravenous fluids with 10% dextrose/0.9% saline mix (with appropriate electrolyte amounts added), ammonia scavengers including sodium benzoate, sodium phenylacetate, or sodium phenylbutyrate, l‐arginine and/or citrulline and renal replacement therapy.^2^ While continuous veno‐venous renal replacement therapy (CRRT) can remove excess ammonia effectively the extracorporeal blood flow is often limited by the CRRT vascath size thereby limiting ammonia removal. Extracorporeal membrane oxygenation (ECMO), particularly in patients with severe cardiac or circulatory failure, can provide higher blood flow rates that enables rapid removal of ammonia through CRRT.^2^

Patients with severe OTC deficiency present with poor feeding, lethargy and seizures. We report a case of a neonate with OTC deficiency who presented with acute hyperammonemia and acute severe cardiac failure. We question whether cardiac failure may be a rare feature of OTC deficiency that so far has been not been reported in the literature.

## 2 CASE REPORT

Informed consent for this publication was obtained from the parents.

A 3528 g male term baby child became lethargic on day three of life after an uneventful birth with reassuring APGAR 8/9 values. He was breast/bottle fed with expressed breast milk for the first 2 days of life. Due to the lethargy he was admitted to the neonatal intensive care unit (NICU) on day three of life. Arterial blood gas analysis on admission to NICU showed a pH of 7.44, pCO2 22, bicarbonate 15 mmol/L and a base excess of −5.9 mEq/L which, after 6 hours of intubation and mechanical ventilation for type 2 respiratory failure worsened to a pH of 7.10, pCO_2_ 55, HCO_3_ 16 mmol/L base excess of −15.7 mEq/L and lactate 8.4 mmol/L. The plasma ammonia level on admission to NICU was 400 μmol/L (normal range < 110 μmol/L for <1 year old, <50 μmol/L for >1 year old). On admission to NICU liver function tests and coagulation tests were normal. A UCD was suspected, and intravenous sodium benzoate and l‐arginine were commenced on day three of life and all feeds were stopped. Urgent amino acid analysis revealed a glutamine level of 3540 μmol/L (normal range, 20‐240) with a citrulline level of less than 2 μmol/L (normal range 5‐33 μmol/L). Urine organic acids showed an orotic acid of 42 mmol/mol creatinine (normal range < 6) confirming a biochemical diagnosis of OTC deficiency on day four. Due to the high lactate level an echocardiogram was done which showed severe left ventricular (LV) dysfunction and dilation (fractional shortening of the LV 8‐13%), however, no abnormal myo‐ or endocardial echo brightness was present. The right ventricular function was normal. Eight hours after starting intravenous sodium benzoate and L‐arginine the plasma ammonia level had increased to 1476 μmol/L. Given the severe LV dysfunction, the rising lactate to 8.4 mmol/L and the need for CRRT, the neck vessels were cannulated and veno‐arterial ECMO (VA‐ECMO: Rotaflow Centrifugal Pump [MAQUET, Germany]) with continuous hemodiafiltration was initiated. While on ECMO, the blood flow through the CRRT circuit was 300 mL/min with a filtration rate of 300 mL/hr and dialysate of 300 mL/hr. Plasma ammonia levels decreased to 170, 120, and 67 μmol/L after 12, 24, and 36 hours of continuous veno‐venous hemodiafiltration (CVVHDF), respectively. The lactate level normalized to <2 mmol/L within 10 hours of ECMO commencement. On ECMO the cardiac function improved from the severe dysfunction seen before ECMO support was initiated to only mild to moderate dysfunction on day two of the ECMO run. After 3 days of ECMO support echocardiography confirmed complete recovery of LV function. He was discharged without any evidence of neurological deficits on day 38. He subsequently had a successful liver transplant at 9 months of age. During assessment by speech‐ergo‐ and physiotherapists at 15 months of age he showed developmental delay with gross motor skills on fifth centile for age. He also demonstrated a delay in language and feeding skills; however, he showed improvement in all three areas over the 12 months prior to the assessment at 15 months of age.

## 3 DISCUSSION

We report successful treatment of a neonate presenting with severe dilated left ventricular dysfunction with acute hyperammonemia caused by OTC deficiency. While treatment of a metabolic crisis due to OTC deficiency with renal replacement therapy is well established, presentations with severe left ventricular dysfunction needing mechanical circulatory support have not been reported to date. In the Paediatric Cardiomyopathy Registry (PCMR) the etiology of dilated cardiomyopathies was unknown in two thirds of cases.^3^ Of the remaining one third 11% (4% of the total group) were caused by inborn errors of metabolism: 46% were caused by mitochondrial disorders, 24% by Barth syndrome and 11% by primary carnitine deficiency, but none were reported to be caused by UCDs.^3^, ^4^ Given dilated cardiomyopathy has never been described in UCD the pathophysiology is hard to determine. Urea cycle disorders present with hyperammonemia as well as increases in orotic acid. In addition, a severe metabolic acidosis is seen in acute crises. Orotic acid has a cardioprotective effect and is not the reason for poor myocardial function in patients presenting with UCD crises.^5^ However, the intracellular lowering of pH, with subsequent effects on intermediary metabolism or oxidization of mitochondrial components may well contribute to the overall impairment of cardiac function. In addition, Bing's hypothesis may explain further why severe LV dysfunction developed: hyperammonemia might play a role in the development of cardiac failure based on the findings that ammonia produces oxidative stress with consequent endoplasmic reticulum (ER) stress and apoptosis. Oxidative stress in turn alters histone deacetylase inhibition, ER stress, autophagy and neurohumoral stimulation.^6^ Phenylbutyrate, which is used in the treatment of UCDs, attenuates ER stress and subsequently decreases pressure‐loaded myocardial hypertrophy in mice.^7^ However, phenylbutyrate was not used for the treatment of this case and can therefore be excluded as the cause for the cardiac dysfunction. It would have been interesting to relate NH4 levels with echocardiographically documented cardiac function, or lactate levels. However, the combination of VA‐ECMO support and CVVHDF makes both variables independent of each other: the NH4 is lowered by CVVHDF, while VA‐ECMO provides supramaximal cardiac support, which makes it impossible to establish a definitive link between the improved cardiac function and the lowered NH4 level.

Acute hyperammonemia is a medical emergency, and aggressive rapid correction of persistent hyperammonemia is recommended.^2^ Peak plasma ammonia levels of more than 500 μmol/L and unresponsiveness to treatment for hyperammonemia are associated with irreversible neurological injury and subsequent death.^8^ Therefore, immediate removal of ammonia using continuous renal replacement therapy is required for neuroprotection when medical drug treatment fails. The efficiency of CVVHDF is limited by the maximum blood flow that can be achieved through the respective vascath used for a given patient.^9^ Veno‐arterial ECMO therapy, by having larger cannulas placed surgically (rather than percutaneously in CVVHDF) allows for a much higher blood flow through the renal replacement therapy circuit, which leads to accelerated removal of plasma ammonia, as well as cardiac support at the same time.^10^ Robinson et al reported successful use of ECMO‐driven hemodialysis with a survival rate of 69% at discharge.^11^

## 4 CONCLUSIONS

Patients with a new diagnosis of OTC deficiency should have an echocardiogram performed to exclude myocardial dysfunction. Severe left ventricular dysfunction with hyperammonemia caused by OTC deficiency (as well as other IEM causing hyperammonemia) can be effectively treated with VA‐ECMO combined with continuous renal replacement therapy.

## CONFLICT OF INTEREST

The authors declare no conflicts of interest.
